# Noninvasive Early Disease Diagnosis by Electronic-Nose and Related VOC-Detection Devices

**DOI:** 10.3390/bios10070073

**Published:** 2020-07-06

**Authors:** Alphus Dan Wilson

**Affiliations:** Pathology Department, Southern Hardwoods Laboratory, Center for Bottomland Hardwoods Research, Southern Research Station, USDA Forest Service, 432 Stoneville Road, Stoneville, MS 38776, USA; dan.wilson2@usda.gov; Tel.: +1-662-336-4809

**Keywords:** early disease detection, clinical pathology, electronic nose, disease diagnostics, point-of-care testing, chemical sensors, disease biomarkers, metabolomics, volatile organic compounds (VOCs), White-nose Syndrome, Emerald ash borer

## Abstract

This editorial provides an overview and summary of recent research articles published in *Biosensors* journal, volumes 9 (2019) and 10 (2020), within the Special Issue “*Noninvasive Early Disease Diagnosis*”, which focused on recent sensors, biosensors, and clinical instruments developed for the noninvasive early detection and diagnosis of human, animal, and plant diseases or invasive pests. The six research articles included in this Special Issue provide examples of some of the latest electronic-nose (e-nose) and related volatile organic compound (VOC)-detection technologies, which are being tested and developed to improve the effectiveness and efficiency of innovative diagnostic methodologies for the early detection of particular diseases and pest infestations in living hosts, prior to symptom development.

## 1. Introduction

The *Biosensors* Special Issue “*Noninvasive Early Disease Diagnosis*” was conceived and initiated to celebrate over 30 years of electronic-sensor devices and technologies developed for disease detection in the field of clinical pathology since electronic-nose (e-nose) devices were first introduced in the mid-1980s. A wide diversity of sensor-system applications, based on e-nose and related volatile organic compound (VOC)-detection technologies, continue to be developed and applied to biomedical, clinical, and diagnostic applications. Among the key advantages of electronic instruments designed to detect complex mixtures of VOC analytes in gaseous clinical samples are the capabilities of achieving noninvasive early disease detection before disease symptoms appear. The capability of classifying and characterizing gaseous chemical samples into relatively simple sensory outputs make e-noses and related electronic devices unique among diagnostic analytical instruments. These devices have the improved characteristics of simple operation, relatively low-cost, good precision, and real-time operation with high sample through-put capabilities. The research articles reviewed here (from this Special Issue) include studies devoted to the common goal of developing improved and effective diagnostic applications, using various types of specialized technologies, which take advantage of changes in host VOC-emissions resulting from physiological changes associated with pathogenesis (during early disease development) and/or internal infestations by destructive pests, such as by nonnative and invasive xylophilous insects that attack commercially-important native forest and plantation trees.

## 2. Review of Special Issue Contributions

The Special Issue, “*Noninvasive Early Disease Diagnosis*”, published in the MDPI journal *Biosensors*, is composed of six research articles, representing the results of original studies conducted by individuals within research teams operating in or associated with five countries, including Australia, Belgium, the Netherlands, the Republic of Indonesia, the United Kingdom, and the United States. The presented works are related to the testing of electronic instruments and associated methodologies being developed for the noninvasive early detection and diagnosis of human and wildlife diseases, as well as infestations of living trees by nonnative insect pests.

The research fields and disciplines (within which the scientists and researchers of the published papers were operating) were used as a basis for categorizing the types of studies included in this Special Issue, including human clinical pathology, veterinary-wildlife pathology, and studies on invasive insect-pest infestations of native forest trees that resulted from new incidental introductions of nonnative insects through U.S. ports of entry. These specified categories were used in the following sectional headings devoted to research studies associated with the corresponding research disciplines.

### 2.1. Human Clinical Pathology

The development of new electronic methods and approaches to improve the speed and accuracy of early diagnoses for gastrointestinal (GI-tract) diseases has been a major area of investigation in human clinical pathology and diagnostic medicine in recent years. Inflammatory bowel disease (IBD), which includes Crohn’s disease (CD) and ulcerative colitis (UC), is a relapsing, chronic condition of unknown etiology causing gut inflammation, leading to abdominal cramping, diarrhea, vomiting, fatigue and weight-loss. Although UC generally only affects the colon (large intestine), CD may affect the entire digestive system. Misdiagnosis can have serious consequences for patients, because delays in correct diagnosis may increase risks of bowel stenosis, requiring intestinal surgery. Early diagnosis of IBD continues to be a clinical challenge because current tests, which include endoscopic investigations and imaging of the lower GI-tract, histological examination, and fecal inflammatory biomarkers, are invasive and costly. Colonoscopy with histology remain the “gold standard” for diagnosing IBD. This procedure is invasive and uncomfortable to patients, generally involves multiple biopsies, and has an associated morbidity.

Tiele et al. [1] evaluated the efficacy of breath analysis for the diagnosis of IBD by examining VOCs in exhaled breath. Breath samples were analyzed using an experimental, in-house built electronic nose (Wolf eNose), and a commercial gas chromatograph–ion mobility spectrometer (G.A.S. BreathSpec GC-IMS). Analysis results from the GC-IMS indicated that detections of decreases in butanoic acid and acetic acid in IBD patients, compared to healthy controls, contributed significantly to the efficacy of IBD detection by GC-IMS breath analysis. These specific VOCs, short-chain fatty acids produced in the colon by fermentation of cellulosic fiber, have recently been identified as important discriminatory biomarker VOC-metabolites for IBD. Wolf eNose sensors sensitive to ammonia, sulfur dioxide, and nitrogen dioxide showed the greatest differences in output responses, contributing most to discriminations between diagnostic groups. The authors concluded that these technologies have some key advantages over conventional diagnostic methods, including being relatively inexpensive, portable, non-invasive, applicable for high-throughputs, and suitable for nearly all patient demographics, including vulnerable patients, such as children and the elderly. Both technologies consistently separated IBD from non-diseased controls.

Another GI-tract disease with symptoms similar to IBD is coeliac disease (CD), which also has great potential for e-nose detection via non-invasive VOC-biomarker analysis. The occurrence of chronic, immune-mediated enteropathy in CD is triggered by ingestion of gluten in genetically-predisposed individuals. Only slightly more than a third of all CD patients display classical symptoms, such as diarrhea, weight loss, and abdominal pain, whereas most patients are asymptomatic or have extra-intestinal symptoms. A small minority of CD patients develop refractory coeliac disease (RCD), of two known types (I and II), with persistent or recurrent villous atrophy, despite adherence to a strict gluten-free diet. Current methods for diagnosing CD consist of serological screening for circulating transglutaminase-2 antibodies, followed by diagnostic confirmation through gastroduodenoscopy, and histological examination of duodenal mucosal biopsies, scored for villous atrophy, crypt hyperplasia, and intraepithelial lymphocytes. An alternative diagnostic approach is through electronic detection and analysis of fecal VOC profiles as potential biomarkers for intestinal disease.

Rouvroye et al. [2] investigated the potential for analyzing the VOC profiles from stool samples of pediatric CD patients and healthy controls by means of GC-IMS, to determine the usefulness of this technique for the differentiation of RCD from CD, and to assess fecal VOCs as potential chemical biomarkers for RCD II. The study revealed no significant differences between fecal VOC-profile patterns of RCD from healthy controls, based on relatively small sample size used in this pilot study. However, CD could be discriminated from RCD and healthy controls based on fecal VOC profiles, which suggested that GC-IMS has potential as a novel tool for the detection of non-invasive VOC biomarkers in stool samples for RCD and CD. The authors planned to conduct additional studies using a larger cohort, to further investigate and validate these findings, prior to their application in clinical practice.

The current standard methods for care of infected skin wounds is to immediately provide empirical treatment with antibiotics and specialized care, following a subjective clinical assessment and swab culture. Antimicrobial therapy usually is based on a best-guess approach for determining the causative organism. The wound is normally swabbed for microscopy and culture over a 48 to 72-h period, to assess the presence of microbial pathogens, and determine the bacterial sensitivity or resistance to initial treatment. However, swab results may be misleading or provide inaccurate or inconclusive results. Both techniques are potentially unreliable and result in delays in using targeted antibiotics. Other disadvantages of current methodology are that the misidentification of the causal agent of a patient’s wound infection may result in the patient receiving incorrect antibiotic therapy, which may result in greater and more severe infections and contribute to antibiotic or antimicrobial resistance. A potential new approach and solution is to detect and monitor VOC-emissions that emanate from skin wounds and dressings that are in contact with the wound.

Daulton et al. [3] utilized a G.A.S. GC-IMS instrument (Dortmund, Germany), developed for a range of industrial applications, to analyze and identify headspace VOCs from wound dressings. The instrument consists of a gas chromatography (GC) column, followed by a drift tube ion mobility spectrometer. The GC column separates headspace VOCs by retention times and the molecules, then sequentially ionized via a tritium source within the IMS unit, are moved along the drift tube by an electric field. This study describes the use of this device to differentiate between VOCs produced by an infected wound vs. colonized wound. The identity of unique mixtures of VOCs within emissions produced by microorganisms present in infected wounds or wound dressings provide indications of the types and presence of microbial pathogens. VOCs from skin wounds are by-products of normal microbial metabolic pathways. Thus, their production is usually dependent on the specific microbial species present. Malodorous wound dressings were collected from patients recruited from plastic surgery, vascular surgery, and diabetic dressings clinics. These were a mix of post-operative wounds and vascular leg ulcers. Wound microbiology swabs were taken and antibiotics commenced as clinically appropriate. The GC-IMS results from wound-dressing samples indicated that the method had a sensitivity of 100%, specificity of 88%, and positive predictive value >90% based on an area under the curve (AUC), 91% of which demonstrated the excellent capability of the method to discriminate between infected and uninfected wounds. The authors concluded that VOC detection using GC-IMS has the potential to serve as a diagnostic tool for the differentiation of infected and non-infected post-operative wounds and ulcers. The method potentially could help facilitate the treatment of wound infections because it is acceptable to patients, non-invasive, portable, reliable, and cost effective.

New diagnostic methods and therapeutic options for care of preterm infants have resulted in improved outcomes, although mortality and morbidity rates due to disease are still high, particularly in children born prematurely (preterm). Early detection and prompt treatment of diseases are often impeded by lack of timely and specific clinical signs and effective diagnostic tools. Currently available methods for disease detection are often painful and invasive, such as venous and lumbar punctures for necrotizing enterocolitis (NEC) and late onset sepsis (LOS) diagnostic workup. The development of noninvasive, early diagnostic tools remains crucial for the optimization of care. Metabolic research has pointed to new potential chemical biomarkers for various medical conditions in both adults and children. The sensitivity of metabolomic approaches to detect subtle alterations in metabolic pathways can, in addition, provide insight into mechanisms underlying various pathophysiological conditions. Analysis of VOCs from fecal samples offers the potential as non-invasive preclinical diagnostic biomarkers for various diseases, including NEC, LOS, and many others. There is a significant need for more information to determine and clarify the effects and influences of clinical and environmental factors affecting gut microbiota composition as well as VOC emissions from these sources and their outcome before this technique can be applied in clinical practice.

Deianova et al. [4] conducted an initial study to investigate the influence of gestational age (GA) and mode of delivery as potential factors affecting fecal VOC patterns in preterm infants born before 30 weeks of gestation. They utilized patients from a multicenter cohort study in nine participating neonatal intensive care units (NICUs) in the Netherlands and Belgium. Postnatal fecal stool samples were collected from diapers by nurses at 7-day intervals, and analyzed by a C-320 electronic-nose device (Sensigent ISS, Baldwin Park, CA, USA). The three sampling study groups included 58 preterm infants, 24 vaginally born, and 34 delivered via C-section. No significant differences were found between the three groups identified at any predefined time point in terms of GA and delivery mode. Only weak potential effects of GA on fecal VOC were found, which is possibly explained by other factors to which very preterm infants were exposed, such as broad-spectrum antibiotics and increased oxidative stress, caused by bronchopulmonary dysplasia and intraventricular hemorrhage. The author’s conclusions were that GA and the mode of delivery are specific variables that did not significantly affect natal VOC fecal-emission compositions, and, therefore, correction for these factors in this population was not warranted when performing fecal VOC analysis within the first few weeks of life. This new information could contribute to methodological guidelines for future VOC research and clinical procedures.

### 2.2. Veterinary-Wildlife Pathology

The reliable and timely detection of infectious wildlife diseases caused by virulent microbial pathogens, particularly those that spread rapidly and are responsible for high mortality in resident wildlife populations, require specialized diagnostic methods that provide early indications of disease processes occurring prior to symptom development. This situation is especially difficult in the case of White-nose Syndrome (WNS), a serious disease of insectivorous bats, caused by the nonnative dermatophytic fungus (*Pseudogymnoascus destructans*, or Pd), which damages bats by several destructive mechanisms, leading to dehydration, starvation, riboflavin-induced dermal and hypodermal necrosis, causing membranous-wing disintegration, systemic physiological shocks, and hypothermia, due to exposure when WNS-affected bats attempt winter feeding outside of Pd-infested caves. The current methods used to conclusively diagnose WNS require invasive methods that usually are performed on dead individuals long after symptom development, which precludes the effective application of therapeutic treatments. The need for new improved methods of early disease detection, prior to symptom development, is essential to provide opportunities to mitigate disease through direct management activities (disease suppression and early therapeutic treatments of infected bats) before bats show disease symptoms, and ultimately to reduce the incidence of bat mortality in cave populations during overwintering within hybernacula.

Doty et al. [5] took a new approach to early WNS diagnosis based on the detection of changes in VOC-emissions from bats as a consequence of physiological changes induced by the disease. In order to test the efficacy of the C-320 portable e-nose (Sensigent ISS, Baldwin Park, CA, USA) they chose for this application, the research team first identified the bat host-derived VOCs that were major components found in complex gaseous mixtures present in clinical air samples collected from whole-bat VOC emissions. In this study, they compared the VOC-profiles and corresponding e-nose smellprint signatures of nine bat species during active (non-torpid) physiological states outside of caves in their summer habitats. Bat smellprint signatures were compared to those of 22 pure, VOC analytical standards representative of five chemical classes. E-nose smellprint patterns were analyzed using principal component analysis (PCA), and other statisical methods that showed effective discrimination between bat species at high levels of statistical significance. These results partially demonstrated the potential efficacy of the portable C-320 e-nose to distinguish between species-specific, bat-derived VOC metabolite emissions as significant components of clinical samples that could be collected from bats in caves for disease diagnosis, prior to symptom development. These results suggest the possible development of a new, more reliable and improved approach and means for early WNS-disease detection, based on e-nose VOC-detection capabilities, compared to the current, more tenuous early-detection capabilities of qPCR based on quantification of Pd-pathogen DNA in swabs from external skin surfaces.

Additional results of this study, providing more details of the chemical evidence to explain differences in metabolic pathways responsible for the generation of unique whole-body VOC-emissions from different insectivorous bat species, were presented in a closely related ancillary report [6]. This additional information provides further indications of the significance that different VOC chemical classes of different molecular weight groups, contribute to differences in specific e-nose smellprint signatures between bat species.

### 2.3. Invasive Insect-Pest Infestations

The application of an electronic-nose device as a potential tool for use in forest pest management to noninvasively detect tree infestations by an invasive, nonnative major insect pest of ash (*Fraxinus* species), was investigated in a study by Wilson et al. [7]. The Emerald ash borer (EAB), a buprestid beetle introduced into the United States of America from Eurasia in the early 1990s, is considered the most costly and destructive nonnative (exotic) insect to threaten the health of North American, European, and Asian ash species for at least the past 25 years. The development of new methods for detecting invisible EAB galleries at early insect-infestation stages would facilitate the more effective planning and implementation of EAB pest-suppression and forest management activities in natural hardwood stands, plantations, and in urban environments. The study examined the efficacy of a dual-technology electronic-nose (e-nose)/gas chromatograph device as a means for the early detection of EAB infestations in green ash trees prior to the appearance of visual symptoms of canopy decline. They compared VOC emissions in sapwood from several different EAB-decline classes.

The results indicated significant differences in VOC sapwood profiles for trees from the four EAB-decline class levels. The types, quantities, and composition of VOC metabolites present in sapwood headspace volatiles varied significantly across sample types. Distinct e-nose smellprint patterns, derived from differences in VOC-composition of headspace volatiles from sapwood cores, were discovered from trees with different levels of EAB infestations (decline classes). Consequently, different smellprint patterns were indicative of unique VOC chemical composition. Specific VOC metabolites were identified as chemical biomarkers of healthy and EAB-infested trees indicative of the health states of individual trees. However, few significant differences in major bark phenolic compounds were found between ash decline classes, using ultra-high-pressure liquid chromatography-mass spectroscopy (UHPLC-MS). The dual-technology e-nose was determined to be effective in discriminating between uninfested and EAB-infested trees based on sapwood VOC emissions. Early e-nose detection of EAB larval feeding in the sapwood of *Fraxinus* species could potentially provide a much-needed tool to aid EAB pest management.

## 3. Conclusions

The publications included in this Special Issue provide new information and insights into ways in which electronic-noses and related VOC-detection devices may be used in the development of improved methods and applications for the noninvasive early detection and diagnosis of diseases and pest organisms, prior to symptom development. This approach affords the opportunity to provide preemptive applications to manage diseases and infestations before significant damage has occurred within affected hosts. Therapeutic treatments ultimately are far more effective and lead to much more rapid patient recovery when such treatments can be applied early, as a result of early disease detection. The high level of detectability of VOC-metabolites (by these electronic devices), released from tissues and in excretory clinical samples, provide great improvements as new diagnostic tools to help accelerate and confirm clinical diagnostic assessments, facilitate prescriptions of early appropriate treatments, and ultimately should provide more favorable prognoses for many diseases.
